# Social Frailty Leads to the Development of Physical Frailty among Physically Non-Frail Adults: A Four-Year Follow-Up Longitudinal Cohort Study

**DOI:** 10.3390/ijerph15030490

**Published:** 2018-03-10

**Authors:** Hyuma Makizako, Hiroyuki Shimada, Takehiko Doi, Kota Tsutsumimoto, Ryo Hotta, Sho Nakakubo, Keitaro Makino, Sangyoon Lee

**Affiliations:** 1 Department of Physical Therapy, School of Health Sciences, Faculty of Medicine, Kagoshima University, Kagoshima 890-8544, Japan; 2 Department of Preventive Gerontology, Center for Gerontology and Social Science, National Center for Geriatrics and Gerontology, Aichi 474-8511, Japan; shimada@ncgg.go.jp (H.S.); take-d@ncgg.go.jp (T.D.); k-tsutsu@ncgg.go.jp (K.T.); ryo-h@ncgg.go.jp (R.H.); sho-n@ncgg.go.jp (S.N.); kmakino@ncgg.go.jp (K.M.); sylee@ncgg.go.jp (S.L.)

**Keywords:** social frailty, physical frailty, longitudinal cohort study, aged

## Abstract

Social frailty domains may play an important role in preventing physical decline and disability. The aim of this study is to examine the impact of social frailty as a risk factor for the future development of physical frailty among community-dwelling older adults who are not yet physically frail. A total of 1226 physically non-frail older adults were analyzed to provide a baseline. Participants completed a longitudinal assessment of their physical frailty 48 months later. Their baseline social frailty was determined based on their responses to five questions, which identified participants who went out less frequently, rarely visited friends, felt less like helping friends or family, lived alone and did not talk to another person every day. Participants with none of these characteristics were considered not to be socially frail; those with one characteristic were considered socially pre-frail; and those with two or more characteristics were considered socially frail. At the four-year follow-up assessment, 24 participants (2.0%) had developed physical frailty and 440 (35.9%) had developed physical pre-frailty. The rates of developing physical frailty and pre-frailty were 1.6% and 34.2%, respectively, in the socially non-frail group; 2.4% and 38.8%, respectively, in the socially pre-frail group; and 6.8% and 54.5%, respectively, in the socially frail group. Participants classified as socially frail at the baseline had an increased risk of developing physical frailty, compared with participants who were not socially frail (OR = 3.93, 95% CI = 1.02–15.15). Participants who were socially frail at the baseline also had an increased risk of developing physical pre-frailty (OR = 2.50, 95% CI = 1.30–4.80). Among independent community-dwelling older adults who are not physically frail, those who are socially frail may be at greater risk of developing physical frailty in the near future. Social frailty may precede (and lead to the development of) physical frailty.

## 1. Introduction

Frailty is a state of being vulnerable to the poor resolution of homoeostasis after a stressor event. It is a consequence of the cumulative damage done to many physiological systems over a lifetime [1]. Older adults with frailty have an increased risk of adverse health outcomes, such as falling, various disabilities, hospitalization and death [2,3,4]. Assessments to identify frailty and intervention strategies to reduce and prevent frailty are therefore important topics in an aging society.

The definition of the physical frailty phenotype is well known; many previous studies have examined its impact on adverse health outcomes [5]. Although frailty is recognized as a multidimensional construct comprising not only physical, but also psychological and social conditions and symptoms [6,7], the psychological and social domains of frailty have not been sufficiently explored [8,9]. In recent years, some researchers have developed indexes of social frailty [10,11] and social vulnerability [12,13] to assess the impact of social frailty on health outcomes. 

Social frailty domains, including social roles, social networks and social activity in older adults, may require higher levels of functioning. A decline in social roles is likely to precede the onset of IADL (intellectual and instrumental activity of daily living) disabilities among community-dwelling independent older adults [14]. Previous studies have demonstrated that low levels of social activity, low participation in social roles and poor social relationships have a negative impact on physical and cognitive function [15,16,17,18]. In addition, loneliness and social isolation have a negative impact on health outcomes. [19] Loneliness is associated with a more rapid motor decline in old age [17]. Social frailty domains may play an important role in preventing physical decline and disability [10,11,20]. However, it is unclear whether social frailty is caused by a physical frailty phenotype.

Examining the longitudinal relationship between physical and social domains may provide information that could help to develop more effective strategies for preventing the multidimensional construct of frailty. This prospective study has examined the impact of social frailty as a risk factor for the future development of physical frailty among community-dwelling older adults who are not yet physically frail.

## 2. Materials and Methods 

### 2.1. Participants

This prospective study has analyzed data drawn from the Obu Health Promotion for the Elderly Study (OSHPE). The OSHPE is a part of a National Center for Geriatrics and Gerontology Study of Geriatric Syndromes (NCGG-SGS), a cohort study whose primary goal was to establish a screening system for geriatric syndromes in the community-dwelling population [21,22].

The present study has analyzed longitudinal data from 1226 community-dwelling older adults ≥65 years (mean age 70.4 ± 4.1 years), who participated in both the first and second waves of the OSHPE and who were not physically frail at the time of the first wave assessment. The first wave of the OSHPE was held between August 2011 and February 2012; during this wave, 5104 community-dwelling elderly people participated in a baseline OSHPE assessment. Among participants who took part in the first wave of the OSHPE, 2834 participated in a second wave OSHPE assessment. This four-year follow-up assessment was held between August 2015 and February 2016.

**Figure 1:**
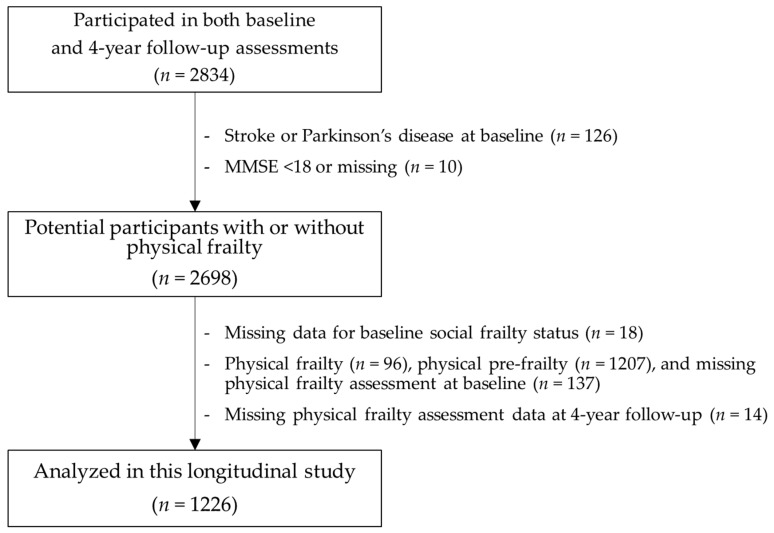
Participant inclusion criteria flow diagram. MMSE: Mini-Mental State Examination.

The present longitudinal study looks at participants who had no physical frailty or pre-frailty at the time of the baseline assessment. Participants with a baseline history of stroke, Parkinson’s disease or cognitive impairment (e.g., Mini-Mental State Examination (MMSE) score [23] < 18) were excluded (Figure 1). These medical conditions can cause disease-based decline, as opposed to age-related decline, and could thus have reduced the reliability and validity of our frailty assessments. Informed consent was obtained from all participants prior to their inclusion in the study. The Ethics Committee of the National Center for Gerontology and Geriatrics approved the study protocol (Ref No. 923).

### 2.2. Physical Frailty

The status of physical frailty is based on the following five conditions: slowness, weakness, exhaustion, low levels of activity and weight loss [2]. Slowness is established using a predetermined cut-off (<1.0 m/s) for comfortable walking speed [24]. Weakness is defined using maximum grip strength and gender-specific cut-offs (<26 kg for men and <18 kg for women) [25]. Exhaustion is present if the participant answers “yes” to the following question from the Kihon Checklist, a self-reported comprehensive health checklist developed by the Japanese Ministry of Health, Labor and Welfare [26]: “In the last two weeks, have you felt tired for no reason?” We evaluated the participants’ levels of physical activity by asking the following questions: (1) “Do you engage in moderate levels of physical exercise or sports aimed at health?” and (2) “Do you engage in low levels of physical exercise aimed at health?” Participants who answered “no” to both questions were classified as having low levels of activity [24]. Participants were assessed as having weight loss if they answered “yes” to the question, “Have you lost 2 kg or more in the past six months?” [26]. Participants with none of these characteristics were not physically frail; those with one or two characteristics were considered physically pre-frail; and those with three or more characteristics were considered physically frail [27]. In this study, we examined physical frailty status at the baseline and at four-year follow-up assessments. Participants who were not physically frail at the baseline were assessed during the four-year follow-up period to see whether they had developed any physical frailty or pre-frailty. 

### 2.3. Social Frailty

To assess and determine the participants’ social frailty, we prepared a questionnaire with seven questions about daily social activities, social roles and social relationships [11]. Five of the seven items in the self-reported questionnaire were significantly associated with disability-related incidents; these were going out less frequently than last year (yes), visiting friends sometimes (no), feeling like helping friends or family (no), living alone (yes) and talking with someone every day (no). Two items (getting bored often and having friends to talk to on the telephone) were not significantly associated with disability-related incidents [11]. The five questions above (used to define social frailty) had a significant association with disability-related incidents in a previous study. Participants with none of these conditions were not frail; those with one condition were considered pre-frail; and those with two or more components were considered frail [11]. The baseline social frailty levels of participants were grouped into the following three categories: not socially frail, pre-fail and frail.

### 2.4. Covariates

Using face-to-face interviews, we examined the participants’ characteristics and medical histories. Each person’s body mass index (BMI) and physical performance, including grip strength and walking speed at baseline, were examined as covariates. The grip strength of each participant’s dominant hand was measured in kilograms, using a Smedley-type handheld dynamometer (GRIP-D; Takei Ltd., Niigata, Japan) [28]. Walking speed was measured in seconds, using a stopwatch. Participants were asked to walk on a flat, straight surface at a comfortable walking speed. Two markers indicated the start and end of a 2.4-m path. Participants walked 2 m before passing the start, so as to be walking at a comfortable pace by the time they reached the timed section of the path [28].

### 2.5. Statistical Analysis

This study used Mantel-Haenszel tests to define the proportion trend and a one-way analysis of variance (ANOVA) for continuous measures to test differences in the baseline characteristics of different groups, based on baseline social frailty status. Incidents related to physical frailty and pre-frailty were calculated for each group during the four-year follow-up assessment. Participants were classified as not socially frail, pre-frail and frail, in accordance with their baseline social frailty status.

The association between baseline social frailty and the development of physical frailty or pre-frailty over four years was examined using multivariate logistic regression analyses. The first model (Model 1) in the multivariate logistic regression analysis was adjusted for age and gender. Model 2 included age, gender, BMI, MMSE, the number of prescribed medications, hypertension, heart disease, diabetes mellitus, osteoporosis and physical frailty status as covariates. The adjusted odds ratios (ORs) for incidents related to physical frailty and/or pre-frailty were estimated with 95% confidence intervals (95% CIs). All analyses were conducted using IBM SPSS Statistics 24.0 (IBM Japan, Tokyo, Japan). The level of statistical significance was set at *p* < 0.05.

## 3. Results

### 3.1. Subsection Characteristics of Participants

The baseline characteristics of the participants and the differences in social frailty between the groups are described in Table 1. Of the 1226 participants who were not physically frail, 250 (20.4%) were considered socially pre-frail and 44 (3.6%) were considered socially frail at the baseline assessment. There were no differences in the baseline characteristics, such as age, gender, medical history, prescribed medications, BMI and grip strength, of groups divided by social frailty status. However, there was a difference in walking speed, with the social frailty group walking at a slower speed (*p* = 0.009).

### 3.2. Associations between Social Frailty and the Development of Physical Frailty

During the four-year follow-up assessment, 24 participants (2.0%) developed physical frailty and 440 (35.9%) developed physical pre-frailty. The rates of developing physical frailty and pre-frailty were 1.6% and 34.2% in the non-frail group, 2.4% and 38.8% in the socially pre-frail group and 6.8% and 54.5% in the socially frail group, respectively.

The results of the multivariate logistic regression analyses are represented in Table 2 and Table 3. The multivariate logistic regression analyses indicated that baseline social frailty significantly increased the risk of physical frailty-related incidents (OR 4.47, 95% CI 1.25–16.06), as well as physical pre-frailty (OR 2.84, 95% CI 1.53–5.29) in crude models. However, baseline social pre-frailty was not significantly associated with an increased risk of physical frailty or with pre-frailty-related incidents. In the adjusted model (Model 2), which included potential covariates, participants who were classified as socially frail at the baseline had an increased risk of developing physical frailty, compared with participants who were not socially frail (OR 3.93, 95% CI 1.02–15.15). Those with baseline social frailty were also at greater risk of developing physical pre-frailty (OR 2.50, 95% CI 1.30–4.80).

## 4. Discussion

The present prospective study has revealed that social frailty can lead to physical frailty in a relatively short period of time among adults who are not physically frail. Among community-dwelling older adults who are not physically frail, those with baseline social frailty have approximately four times the risk of experiencing an incident related to physical frailty as those who are not socially frail, even after controlling for several potential covariates.

Some recent studies have focused on including or developing social domains to assess frailty [4,10,11,29,30]. This study identified the social frailty status of participants using five simple questions: participants with two or more conditions were considered socially frail. Although it may be useful to include other items in the social domain, such as economic status [31] and lifetime occupation [32], this very simple index has been shown to predict an increased risk of disability-related incidents [11]. These simple questions are thus a very useful tool for assessing social frailty.

On the other hand, physical frailty may have a greater impact than social frailty on the incidence of disability. In fact, physical frailty is associated with a greater risk of disability than social frailty in our cohort database [11,33]. If social frailty is one of a range of important factors that lead patients who are not frail to develop physical frailty, it is essential to assess social frailty to understand the future risk of poor health outcomes. This prospective study shows that participants with baseline social frailty are approximately four-times more likely to have an incident related to physical frailty in four years than participants who are not socially frail. Although both socially frail and non-frail groups had relatively fast walking speeds (>1.25 m/s), participants with social frailty had slower baseline walking speeds than those without. On the other hand, there was no difference in grip strength. Age-related changes in mobility may be related to increasing social frailty. Older adults who are socially frail, even if they are not physically frail, may be potential targets for disability prevention.

Social aspects of the lives of older adults, including social connectedness, social relations, social engagement and the social environment may impact the extent to which physical functions decline and physical inactivity increases [34,35,36,37]. Our previous cross-sectional analysis revealed that older adults with social frailty had lower physical function than those without [20]. Although these social factors may play a role in maintaining good health, including physical functions, few studies have used the concept of frailty to explore the longitudinal relationships between the social and physical domains. The current study has an important clinical message: social frailty leads to the development of physical frailty among adults who are not physically frail. Assessing social frailty and implementing intervention strategies to avoid social frailty may be useful for preventing physical frailty and disability-related incidents.

The current study has focused on the relationship between baseline social frailty and incidents caused by physical frailty. Older adults’ social domains may have a positive impact on their cognitive function. Previous studies have indicated that poor social relationships, low levels of social activity and low social engagement are associated, not only with cognitive decline [38,39], but also with an increased risk of dementia [40,41,42]. As several issues in each domain, including the definition of frailty, need to be further explored, future studies should analyze the relationships between the various domains, including cognitive impairment.

One essential message is that social frailty is reversible [43]. Interactive and social activities, such as having conversations and going outdoors, increase stimulation of the brain [44,45]. Participating in a resident-centered community intervention program called a ‘community salon’ may also be useful for preventing functional disabilities [46]. Future research should examine whether reducing social frailty by increasing social activity, social engagement and social relationships can help to prevent physical and cognitive frailty.

This study has several limitations. It has not examined adverse relationships or the longitudinal association between baseline physical frailty and future social frailty. Some older adults can expand their life-space through increased social activity, even when they suffer from physical frailty. Interactive associations between the various domains of frailty are needed to develop effective prevention strategies in the community. In addition, this study has not assessed the participants’ medical histories or hospital admissions during the follow-up period. Illness and hospitalization may accelerate both the physical and social aspects of age-related functional decline.

## 5. Conclusions

Among independent community-dwelling older adults who are not physically frail, those who are socially frail may be at greater risk of developing physical frailty in the near future. Social frailty may precede (and lead to the development of) physical frailty.

## Figures and Tables

**Table 1:** Baseline characteristics of the participants, mean ± SD or %.

Variable	Social Non-Frailty (*n* = 932)	Social Pre-Frailty (*n* = 250)	Social Frailty (*n* = 44)	*p* ^a^
Age, mean ± SD (years)	70.4 ± 4.1	70.4 ± 4.0	71.3 ± 5.8	0.340
Women, *n* (%)	482 (51.7%)	126 (50.4%)	25 (56.8%)	0.871
Medical history, *n* (%)				
Hypertension	353 (37.9%)	99 (39.6%)	18 (40.9%)	0.541
Heart disease	138 (14.8%)	39 (15.6%)	4 (9.1%)	0.654
Diabetes mellitus	86 (9.2%)	24 (9.6%)	4 (9.1%)	0.914
Osteoporosis ^b^	91 (9.8%)	32 (12.8%)	2 (4.5%)	0.785
Prescribed medications, mean ± SD (number)	1.5 ± 1.7	1.7 ± 2.0	1.8 ± 1.5	0.191
BMI, mean ± SD (kg/m^2^)	23.3 ± 3.0	23.3 ± 3.1	23.3 ± 3.0	0.959
Physical performance				
Grip strength, mean ± SD (kg)	29.0 ± 7.4	28.5 ± 7.5	28.1 ± 7.4	0.567
Walking speed, mean ± SD (m/s)	1.32 ± 0.17	1.30 ± 0.17	1.25 ± 0.16	0.009

SD, standard deviation; BMI, body mass index; ^a^ Mantel-Haenszel test for proportion trends and one-way analysis of variance for continuous measures; ^b^ missing values for osteoporosis (n = 1).

**Table 2:** Odds ratios for the development of physical frailty after four years, with reference to baseline social frailty status among older adults without physical frailty.

Baseline Status of Social Frailty	Dependent Value: Incidence of Physical Frailty					
	Crude		Model 1		Model 2	
	OR	95% CI	OR	95% CI	OR	95% CI
Not socially frail	1	[Reference]	1	[Reference]	1	[Reference]
Socially pre-frail	1.50	0.58–3.92	1.49	0.57–3.90	1.22	0.45–3.25
Socially frail	4.47 *	**1.25–16.06**	3.98 *	**1.09–14.59**	3.93 *	**1.02–15.15**

Note: OR, odds ratio; CI, confidence interval; the bold typeface indicates statistical significance; * *p* < 0.05; Model 1: adjusted for age and gender; Model 2: adjusted for age, gender, BMI, number of prescribed medications, hypertension, heart disease, diabetes mellitus, osteoporosis, grip strength (baseline) and walking speed (baseline).

**Table 3:** Odds ratios for the development of physical frailty or pre-frailty after four years, with reference to baseline social frailty status among older adults without physical frailty.

Baseline Status of Social Frailty	Dependent Value: Incidents Related to Physical Frailty or Pre-Frailty					
	Crude		Model 1		Model 2	
	OR	95% CI	OR	95% CI	OR	95% CI
Not socially frail	1	[Reference]	1	[Reference]	1	[Reference]
Socially pre-frail	1.26	0.94–1.67	1.27	0.95–1.69	1.17	0.87–1.58
Socially frail	2.84 **	**1.53–5.29**	2.75 **	**1.46–5.19**	2.50 **	**1.30–4.80**

Note: OR, odds ratio; CI, confidence interval; the bold typeface indicates statistical significance; ** *p* < 0.01; Model 1: adjusted for age and gender; Model 2: adjusted for age, gender, BMI, number of prescribed medications, hypertension, heart disease, diabetes mellitus, osteoporosis, grip strength (baseline) and walking speed (baseline).
